# Efficacy of Normalisation of Advance Care Planning (NACP) for people with chronic diseases in hospital and community settings: a quasi-experimental study

**DOI:** 10.1186/s12913-021-06928-w

**Published:** 2021-09-01

**Authors:** Sarah Jeong, Peter Cleasby, Se Ok Ohr, Tomiko Barrett, Ryan Davey, Christopher Oldmeadow

**Affiliations:** 1 School of Nursing and Midwifery, University of Newcastle, 10 Chittaway Road, Ourimbah, NSW 2258 Australia; 2 Division of Aged, Subacute and Complex Care, PO Box 6088, Central Coast Local Health District, Long Jetty, NSW 2261 Australia; 3 Hunter New England Nursing and Midwifery Research Centre, Hunter New England Local Health District, James Fletcher Campus, Gate Cottage, 72 Watt St, Newcastle, NSW 2300 Australia; 4 Department of Aged Care Services, Wyong Hospital, PO Box 4200, Central Coast Local Health District, Lakehaven, NSW 2263 Australia; 5 Hunter Medical Research Institute, Lot 1, Kookaburra Circuit, New Lambton Heights, Newcastle, NSW 2305 Australia

**Keywords:** Advance care planning, Advance care directives, Hospital, Community, Nurses

## Abstract

**Background:**

Advance Care Planning (ACP) has emerged to improve end-of-life processes and experiences. However, the available evidence presents the gloomy picture of increasing number of older people living with chronic diseases and the mismatch between their preferences for and the actual place of death. The study aimed to investigate the efficacy of normalisation of an Advance Care Planning (NACP) service delivered by specially trained Registered Nurses (RNs) in hospital and community settings.

**Methods:**

A quasi-experimental study was conducted involving 16 sites (eight hospital and eight community sites) in Australia. Patients who were aged ≥18 years, who had at least one of nine chronic conditions, and who did not have an Advance Care Directive (ACD) were offered the NACP service. ACP was normalised as part of routine service on admission. The intervention, NACP, was a series of facilitated conversations about the components of ACP. The primary outcomes which included the completion of ACDs, and/or appointment of an Enduring Guardian (EG), were assessed in both intervention and control sites at pre and post intervention stages. Numbers of patients who completed an ACD or appointed an EG were described by count (percentage). ACD completion was compared between intervention and control sites using a logistic mixed effects regression model. The model includes fixed effects for treatment group, period, and their interaction, as well as random site level intercepts. Secondary model included potentially confounding variables as covariates, including age, sex and chronic diseases.

**Results:**

The prevalence of legally binding ACDs in intervention sites has increased from five to 85 (from 0.85% in pre to 17.6% in post), whereas it has slightly decreased from five to 2 (from 1.2% in pre and to 0.49% in post) in control sites (the difference in these changes being statistically significant *p* < 0.001). ACD completion rate was 3.6% (*n* = 4) in LHD1 and 1.2% (*n* = 3) in LHD2 in hospital whereas it was 53% (*n* = 26) in LHD1 and 80% (*n* = 52) in LHD2 in community.

**Conclusions:**

The study demonstrated that NACP service delivered by ACP RNs was effective in increasing completion of ACDs (interaction odds ratio = 50) and was more effective in community than hospital settings. Involvement of various healthcare professionals are warranted to ensure concordance of care.

**Trial registration:**

The study was retrospectively registered with the Australian New Zealand Clinical Trials Registry (Trial ID: ACTRN12618001627246) on 03/10/2018. The URL of the trial registry record http://www.anzctr.org.au/trial/MyTrial.aspx

**Supplementary Information:**

The online version contains supplementary material available at 10.1186/s12913-021-06928-w.

## Background

People are living longer than ever before, but the question of whether they are living better remains. More importantly, is their experience of dying consistent with their views on life and death? Are they dying well or better than how they have lived? The available evidence suggests the gloomy picture of how people live and die. Globally, 58% of people aged 65 years and over live with two or more chronic diseases (e.g. cardiovascular diseases, cancer, respiratory diseases and diabetes), which represent a major disability burden while living [1], and these are the leading causes of deaths [2]. Whist 70–80% of people in Australia indicated that they wish to die at home [3], 80% of all deaths occur in hospitals or residential aged care facilities [4]. Given the increasing number of people with chronic diseases worldwide, and the considerable financial and emotional costs associated with unwanted, unnecessary or contested medical treatment at the end of life, it is essential to engage in conversations about how and where people die, and to improve end-of-life care services. Advance Care Planning (ACP) has emerged to do exactly that, ‘to improve end-of-life processes and experiences’.

Advance Care Planning (ACP) is a process where patients discuss their values in life and goals for health care, and future health care preferences with family members for a time when they are not able to make health care decisions [5]. There is a lack of consensus about what ACP entails in international literature [6] and there are variations in jurisdictional legislation [7]. According to the Ministry of Health in New South Wales (NSW), Australia, there are two main elements in ACP; the written Advance Care Directive (ACD) and the appointment of an Enduring Guardian (EG) or nomination of Person Responsible (PR) as a substitute decision maker (SDM). It is ideal if ACDs are documented as an outcome of ACP when the person is still well and capable of making decisions [5]. For an ACD to have sufficient authority to act on, in other words, to be legally binding, four conditions of relevance, specificity, competence and currency should be satisfied [5, 8].

The benefits of ACP to patients and their families are well documented [9]. Patients consistently report greater satisfaction with their health care and a more positive outlook on end of life care [10, 11]. Treating health professionals report increased satisfaction with the implementation of an ACD as they can respect patient wishes [11, 12]. ACP can also help remove the burden of decision making from family members, at a particularly difficult time in their lives [13, 14]. Although significant work has been done to promote ACP in Australia and worldwide, a low to very low uptake of ACP is commonly reported [15–22]. There are numerous reasons for the lack of ACP uptake in Australia. Regardless of cultural and ethnic backgrounds, reasons for poor uptake were shown to stem from a lack of understanding of the concept. Use of numerous forms and different medical terms are often seen as confusing and time consuming [20]. Healthcare professionals also reported that they lacked enough education on the matter. They felt underprepared and lacked confidence to help patients with their ACP process [13, 14, 20, 23, 24].

Previous studies clearly point out that the challenges in ACP lie in the processes of: 1) initiation of conversation; 2) discussion of important issues; 3) documentation of the wishes; 4) storage of the documented wishes; and 5) access and execution of the written wishes. This is attributed to the fact that ideally ACP involves an interactive discussion between the patients, their SDM and their healthcare professionals. It allows the patient to articulate their life values and expectations for both life and future medical care. ACP also provides patients with increased awareness of their treatment options so they are able to make more informed decisions [23]. It should also include discussions that focus on the general understanding of disease trajectories and prognosis [24]. Obviously, coordination of multiple meetings between all the stakeholders, takes time. For service provision and delivery, it is imperative for policy makers and healthcare professionals to know how long ACP processes may take, and how many people will complete ACD at the end of the process, given that not all discussions during ACP may result in documentation of ACD. Current literature provides little insight on these issues.

The aim of the study was to investigate the efficacy of normalised ACP (NACP) service by four specially trained ACP Registered Nurses (RNs) for people with chronic diseases in uptake of ACP service and completion of ACDs in hospital and community settings. The secondary aim of the study was to identify factors that influence the completion of ACP in hospital and community health settings.

## Methods

A quasi-experimental study was conducted to address the challenges in ACP processes as identified above and the study protocol is reported elsewhere [25].

### Study design

This study employed a quasi-experimental design with two non-randomised groups; 1) intervention sites with ACP RNs, and 2) control sites without ACP RNs.

### Study setting

The study involved 16 sites (eight hospital and eight community) across two Local Health Districts (LHDs) in NSW, Australia. The eight sites in each health district were then pair-matched into four control and four intervention sites based on admission rates, patient profile, number of deaths per month/year, average length of stay and number of referrals from/to hospital and community. To minimise potential contamination of intervention the sites were geographically separated. Both public funded and non-government organisation (NGO) community sites were involved in this research, to maximise the generalisability.

### Participants

Selection of eligible patients included all the following criteria:

- Patient aged ≥18 years, and
- Admitted to the wards or receiving community service in participating sites during the 6-month intervention period, and
- Identified in Medical Records as having at least one of the chronic health condition(s) (defined as Cancer, Chronic Kidney Disease (CKD), Chronic Obstructive Pulmonary Disease (COPD), Congestive Heart Failure (CHF), Coronary Artery Disease (CAD), Dementia, Diabetes, Frailty and Hypertension), and
- Patient does not currently have an ACD.

The exclusion criteria included:

- Women who are pregnant and the human foetus
- Children and/or young people (< 18 years)
- People highly dependent on medical care
- People who are experiencing acute severe physical illness and/or an acute episode of mental illness (a diagnosis of anxiety alone may not exclude participation).

**Fig. 1:**
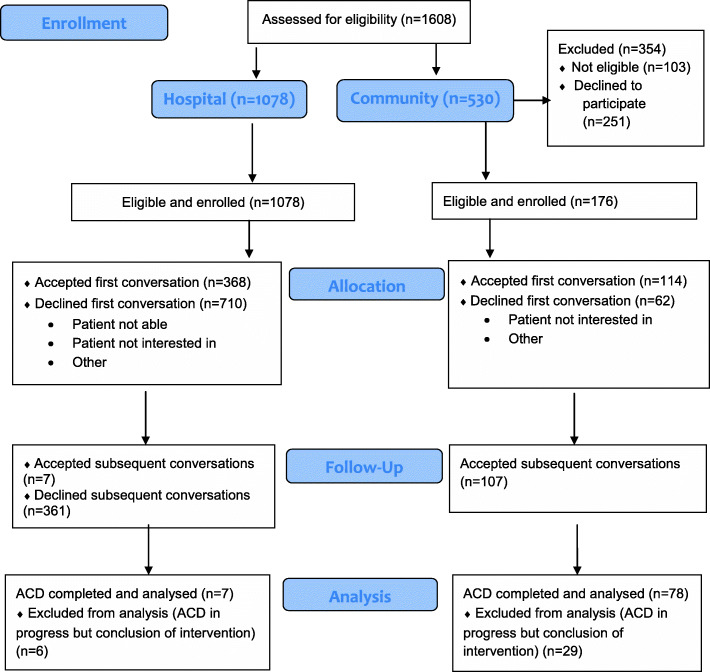
Diagram of the flow of participants in intervention sites

Once potential participants were identified as meeting the inclusion criteria, a robust screening process was applied by admitting medical officer or admitting RN to establish patients’ mental capacity and ability to give valid informed consent using Montreal Cognitive Assessment (MOCA) and Mini-Mental State Examination (MMSE) [25]. Figure 1 presents a diagram of the flow of participants in this study using Consolidated Standards of Reporting Trials (CONSORT flow Diagram) [26].

### Intervention

The intervention used in this study was informed by existing evidence from the literature and clinical experiences over the last 10 years; these concluded that use of ACP by a designated person and using the patient’s own language is the optimal implementation strategy [25, 27–29].

ACP service was normalised as part of routine service on admission. It means that all newly admitted patients who met the inclusion criteria, and who deemed to have mental capacity and ability to give valid informed consent were offered ACP service as part of normalised/routine practice. They could either decline or accept the ACP service just like they could do for a pastoral care service. All patients agreeing to engage with the ACP service during the period of the intervention constituted the sample for the study. ACP RNs, who were not part of the research team but were part of the care team, assessed patients for cognitive impairment and acute episode of mental illness. A diagnosis of dementia and/or anxiety did not exclude participation. The intervention, NACP, was a series of facilitated conversations about the components of ACP between patients, family members and ACP RNs using 1) the one-page ACP Brochure (produced by The NSW Ministry of Health - Making your wishes known), 2) the ACD form in The NSW Ministry of Health Ministry ‘Making an ACD’, and 3) a Conversation Card developed by the research team (a business card size when folded and can be carried in the participating client’s wallet/purse to alert ambulance officers and other treating health care professionals of the existence and location of completed ACDs). Supplementary file 1 provides the graphic picture of these materials used. How the conversation was initiated, and how the subsequent conversations continued are reported in the study protocol [25], and depended on individual patients’ and family members’ responses and situations. Each ACP RN was allocated to split their time between either two hospital wards or two community health service providers for the six-month intervention period, which included 2 weeks of training.

### Outcome measures

The primary outcomes were the completion of ACD (‘Making an ACD’ by NSW Ministry of Health Ministry), and an appointment of Enduring Guardian (EG). Supplementary file 2 provides the definitions of ACD and EG. The secondary outcomes, factors that influence the completion of ACP, were drawn from the primary outcomes.

### Sample size

Power calculations were based off conservative assumptions that 5% of participants at control sites would have a completed NSW ACD, and an intra-class correlation of 0.2. With 16 sites, eight of which randomised to receive the intervention and eight to receive usual care, an average sample size of 24 patients per site would give the study 80% power to detect an absolute increase of 10% between intervention and control sites, with a Type 1 error rate of 5%. This sample size was deemed feasible with the average admittance rate of approximately 10 patients per week.

### Ethics

This project has been approved by the Hunter New England Human Research Ethics Committee (Approval No. 17/12/13/4.16). The study was conducted in accordance with the National Health and Medical Research Council’s National Statement on Ethical Conduct in Human Research (2007), and under the governance of the Human Research Ethics Committee (HREC) at the University of Newcastle and the two Local Health Districts. Informed consent was sought and obtained for uptake of NACP service, and voluntary participation was ensured.

### Data collection

A medical record audit to examine the evidence of ACP was conducted before and during the six-month intervention period at the 16 sites, to investigate the effect of the NACP service delivered by the four ACP RNs. Demographic characteristics of participants such as patient age, gender, and type of chronic health condition were also collected. The details of medical audit process and results of pre-intervention are reported elsewhere [30].

### Data analysis

Participant demographic characteristics (i.e. age, gender, chronic health condition) are summarised between groups (either intervention vs control: LHD1 vs LHD2; or setting: hospital vs community).

Conversation times were aggregated within patients, and summarised as means. Where the time of a conversation was missing, the value was imputed using the mean of the other conversations from that particular site.

Variables are described by mean (standard deviation) if normally distributed and by median (interquartile range) otherwise. Categorical variables such as number of patients who elected to complete an ACD were described by count (percentage).

The primary outcome (ACD completion) was compared between intervention and control sites using a logistic mixed effects regression model. The model includes fixed effects for treatment group, period, and their interaction, as well as random site level intercepts. Secondary model included potentially confounding variables as covariates, including age, sex and chronic diseases.

For the exploratory analysis aims, a logistic regression model was used to assess characteristics associated with ACD completion, crude and adjusted odds ratios from this model are reported together with 95% confidence intervals and *p*-values. The c-statistic and Hosmer-Lemeshow test were also reported to assess goodness of fit.

All tests of significance were set at 5%. Statistical analysis was performed using R version 4.0.1 [31].

### Patient and public involvement

Public involvement was sought at various stages of this study. Senior people were invited to ACP information sessions through local social Seniors’ Clubs in two districts between 2012 and 2016. Attendees shared their understandings, experiences, challenges and suggestions for ACP. The senior people who participated in the sessions identified the challenges in ACP process and this significantly contributed to the research aim and design of the intervention. The outcomes of the engagement with seniors indicated that; 1) routinisation of ACP, 2) by a designated person, 3) about what matters to them, and 4) using their own language should work. The intervention of NACP service was provided to individual participants in the form of completed ACDs with the facilitation of conversations by ACP RNs. Results of the study cannot be directly shared with participants as only de-identified data were collected. All sites involved in the study have been provided with de-identified site specific and aggregated reports, which aimed to inform future ACP service and policy at sites.

## Results

Table 1 presents the demographic characteristics of the participants. The total number of participants enrolled in this study was 1897 at all sites. The mean age was 74.9 years old and male accounted for 48%. The participants (*n* = 1897) reported to have 3894 chronic conditions with the mean 2.1 chronic conditions. The most common chronic condition reported was Hypertension (28.7%) followed by Diabetes (27.7%).

**Table 1:** Demographic characteristics of participants

	LHD1				LHD2				Total
	Pre		Post		Pre		Post		
	INT	CONT	INT	CONT	INT	CONT	INT	CONT	
N	303	226	159	194	278	199	323	215	1897
Mean age years (SD)	75.6 (12.78)	76.6 (14.28)	74.7 (8.98)	78.5 (12.56)	71.8 (14.67)	73.7 (12.98)	77.6 (9.61)	70.8 (16.67)	74.9
Sex (M:F) %	47: 53	47: 53	46: 54	50: 50	48: 52	48: 52	50: 50	51: 49	48: 52
^a^**Chronic conditions N (%, SD)**									
CAD	65 (13.4)	38 (8.4)	36 (12.7)	36 (8.5)	53 (8.7%)	33 (7.2)	67 (9.9)	40 (8.1)	368 (9.5, 12.85)
Cancer	14 (2.9)	44 (9.8)	25 (8.6)	37 (8.7)	50 (8.2)	60 (13)	67 (9.9)	75 (15.2)	372 (9.6, 19.5)
CHF	44 (9.1)	54 (12)	20 (6.9)	58 (13.6)	52 (8.5)	41 (9)	38 (5.6)	49 (9.9)	356 (9.1, 11.2)
CKD	5 (1)	20 (4.5)	5 (1.7)	16 (3.8)	6 (1)	41 (9)	0 (0)	50 (10.1)	143 (3.7, 17.2)
COPD	44 (9.1)	41 (9)	20 (6.9)	41 (9.5)	62 (10.1)	31 (6.7)	67 (9.9)	36 (7.3)	342 (8.8, 14.44)
Dementia	6 (1.2)	24 (5.3)	0 (0)	25 (5.8)	14 (2.2)	14 (3)	0 (0)	14 (2.8)	97 (2.5, 9.01)
Diabetes	186 (38.4)	91 (20)	92 (32.2)	86 (20.1)	211 (34.4)	119 (26)	180 (26.5)	112 (22.7)	1077 (27.7, 46.6)
Frailty	0 (0)	0 (0)	0 (0)	0 (0)	6 (1)	2 (0.5)	11 (1.6)	3 (0.6)	22 (0.6, 3.7)
Hypertension	120 (24.7)	140 (31)	89 (31)	128 (30)	159 (25.9)	117 (25.6)	249 (36.6)	115 (23.3)	1117 (28.7, 45.47)
Total	484 (100)	452 (100)	287 (100)	427 (100)	613 (100)	458 (100)	679 (100)	494 (100)	3894 (100)
Mean	1.6	2	1.8	2.2	2.2	2.3	2.1	2.3	2.1

^a^Participants have multiple comorbidities and the sum exceeds the total number of patients. *INT* Intervention, *CONT* Control, *CKD* Chronic Kidney Disease, *COPD* Chronic Obstructive Pulmonary Disease, *CHF* Congestive Heart Failure, *CAD* Coronary Artery Disease

### Primary outcome: completed ACDs and appointed EGs

Table 2 presents the number of completed ACDs and appointed EGs at intervention and control sites. In the pre-intervention period, a total of 1006 patients’ medical records were audited across eight intervention and eight control sites in LHD1 and LHD2. There was a very low number of ACDs found across both LHDs. In audited records at intervention sites the prevalence of legally binding ACDs was 0.85% (*n* = 5 out of 587) and 1.20% (n = 5 out of 419) at control sites. The number of people who appointed EG was 33 (5.6%) at intervention sites and six (1.43%) at control sites.

**Table 2:** The number of completed ACDs and appointed EG at intervention and control sites

Site	Pre (*n* = 1006)			Site	Post (*n* = 891)		
	ACD Legally binding	ACD Legally nonbinding	EG		ACD Legally binding	ACD Legally nonbinding	EG
**Intervention**							
	Pre (*n* = 581)				Post (*n* = 482)		
LHD1 IH1	0	1	4	LHD1 IH1	3	0	0
LHD1 IH2	1	1	13	LHD1 IH2	1	0	0
LHD1 IC1	0	0	9	LHD1 IC1	11	0	8
LHD1 IC2	0	0	0	LHD1 IC2	15	0	9
LHD2 IH1	0	2	1	LHD2 IH1	1	0	0
LHD2 IH2	3	7	5	LHD2 IH2	2	0	1
LHD2 IC1	1	0	1	LHD2 IC1	36	0	11
LHD2 IC2	0	0	0	LHD2 IC2	16	0	8
**Total**	**5**	**11**	**33**	**Total**	**85**	**0**	**37**
**Control**							
	Pre (*n* = 425)				Post (*n* = 409)		
LHD1 CH3	0	0	0	LHD1 CH3	0	0	0
LHD1 CH4	0	0	1	LHD1 CH4	1	0	0
LHD1 CC3	0	0	0	LHD1 CC3	0	0	0
LHD1 CC4	0	0	0	LHD1 CC4	0	0	0
LHD2 CH3	3	1	1	LHD2 CH3	0	0	1
LHD2 CH4	1	6	2	LHD2 CH4	0	0	0
LHD2 CC3	1	0	2	LHD2 CC3	1	0	2
LHD2 CC4	0	0	0	LHD2 CC4	0	0	0
**Total**	**5**	**7**	**6**	**Total**	**2**	**0**	**3**

Note: *IH* Intervention Hospital, *IC* Intervention Community, *CH* Control Hospital, CC Control Community, *ACD* Advance Care Directives, *EG* Enduring guardian

In the post-intervention period, we audited 891 patients’ medical records. A very low number of ACDs were found in control sites across both LHDs. The prevalence of legally binding ACDs in control sites was 0.49% (*n* = 2 out of 409), which was a slight decrease from 1.20% (*n* = 5) in the pre-intervention period (corresponding to an odds ratio of 0.45). In intervention sites, the overall completion of legally binding ACDs has increased from five to 85 (0.85 to 17.6%), corresponding to an odds ratio of 24.9. The relative change in the odds of this outcome from pre to post for intervention sites was approximately 55 times more than what was recorded at control sites (*p* < 0.0001), which was statistically significant at the 5% threshold. After adjusting for age and sex, and chronic conditions of patients, the difference remained statistically significant (interaction OR = 420; *p* < 0.0001). Analysis of intervention vs control in the post period only indicated that the intervention sites had ‘odds of ACD completion’ approximately 50 times higher than that at the control sites (p < 0.0001). The prevalence of appointment of EG in control sites was 0.73% (*n* = 3 out of 409), which was a slight decrease from 1.43% (*n* = 6) in the pre-intervention period. In intervention sites, the appointment of EG has increased from 33 to 37 (5.62 to 7.68%).

In the pre-intervention period, eight out of 10 ACDs were found in the records of patients admitted to hospital sites. The prevalence of ACDs in hospital settings in LHD1 and LHD2 was 0.1% (*n* = 1) and 0.9% (*n* = 9) respectively. The prevalence of ACDs in the community setting in LHD2 was 0.2% (*n* = 2) and no ACD was found in the community setting in LHD1. In the post intervention period, the number of ACDs completed in hospital settings has increased from one to four (300% increase) in LHD1 and has remained same at three in LHD2. The number of ACDs completed in the community setting has increased from 0 to 26 (2600% increase) in LHD1 and from one to 52 (5100% increase) in LHD2.

For the exploratory aim, multivariable logistic regression results indicated that patients with CAD, Cancer, Diabetes, and Frailty were more likely to have completed an ACD, while patients with CKD, CHF and Dementia were less likely (Table 3). The model fits well to the data (AUC = 0.90, and Hosmer-Lemeshow *p* value = 0.05).

**Table 3:** Multivariable logistic regression between ACD completion and demographic variables

Dependent: ACD		Absent	Present	OR (univariable)	OR (multivariable)
age	Mean (SD)	75.1 (13.7)	77.2 (8.8)	1.01 (1.00–1.03, *p* = 0.140)	1.02 (1.00–1.04, *p* = 0.070)
sex	F	933 (94.4)	55 (5.6)	•	•
	M	865 (95.2)	44 (4.8)	0.86 (0.57–1.29, *p* = 0.478)	0.79 (0.49–1.28, *p* = 0.346)
Coronary Artery Disease	No	1508 (98.7)	20 (1.3)	•	•
	Yes	290 (78.6)	79 (21.4)	20.54 (12.63–34.96, *p* < 0.001)	9.10 (3.96–21.23, *p* < 0.001)
Cancer	No	1511 (98.2)	27 (1.8)	•	•
	Yes	287 (79.9)	72 (20.1)	14.04 (8.97–22.59, *p* < 0.001)	2.98 (1.50–6.05, *p* = 0.002)
Congestive Heart Failure	No	1490 (96.9)	48 (3.1)	•	•
	Yes	308 (85.8)	51 (14.2)	5.14 (3.40–7.78, p < 0.001)	0.28 (0.14–0.52, *p* < 0.001)
Chronic Kidney Disease	No	1631 (94.9)	88 (5.1)	•	•
	Yes	167 (93.8)	11 (6.2)	1.22 (0.61–2.23, *p* = 0.545)	0.39 (0.17–0.85, *p* = 0.022)
COPD	No	1529 (98.2)	28 (1.8)	•	•
	Yes	269 (79.1)	71 (20.9)	14.41 (9.24–23.08, *p* < 0.001)	2.17 (0.98–5.14, *p* = 0.066)
Dementia	No	1705 (94.7)	95 (5.3)	•	•
	Yes	93 (95.9)	4 (4.1)	0.77 (0.23–1.90, *p* = 0.619)	0.13 (0.03–0.40, *p* = 0.001)
Diabetes	No	825 (99.6)	3 (0.4)	•	•
	Yes	973 (91.0)	96 (9.0)	27.13 (10.16–110.66, *p* < 0.001)	4.54 (1.27–21.99, *p* = 0.033)
Frailty	No	1788 (95.3)	88 (4.7)	•	•
	Yes	10 (47.6)	11 (52.4)	22.35 (9.19–55.02, *p* < 0.001)	10.66 (3.27–39.96, *p* < 0.001)
Hypertension	No	782 (99.4)	5 (0.6)	•	•
	Yes	1016 (91.5)	94 (8.5)	14.47 (6.49–41.19, *p* < 0.001)	1.83 (0.63–6.37, *p* = 0.300)

Number in data frame = 1897, Number in model = 1897, Missing = 0, C-statistic = 0.895, H&L = Chi-sq(8) 15.72 (*p* = 0.05)

### Intervention measures: ACP services offered, ACDs and conversation cards completed

NACP service was offered to a total of 1608 new admissions who met the inclusion criteria for 6 months (July – Dec 2018 in LHD1 and Nov 2018 – April 2019 in LHD2) at eight intervention sites across two LHDs (See Table 4). Overall, 482 (30%) patients consented to have the first conversation with four ACP RNs. ACD completion rate was 3.6% (*n* = 4) in LHD1 and 1.2% (*n* = 3) in LHD2 in hospital settings. In community settings, ACD completion rate was 53% (*n* = 26) in LHD1 and 80% (*n* = 52) in LHD2. Each patient who had completed ACDs had an average 3.3 conversations. Each conversation took average 80.77 min. Each completed ACD took average 4.44 h of the ACP RNs’ time spaced around 4 to 6 weeks apart. Conversation Cards were completed and held by all those who completed ACDs (*n* = 85). Another 35 patients commenced a Conversation Card and were in progress at the conclusion of 6-month intervention period.

**Table 4:** A summary of ACP services offered, and ACDs and Conversation Cards completed

ACP service offered	LHD1 Intervention July – Dec 2018			LHD2 Intervention Nov 2018 – April 2019			Total
	Hospital	Community		Hospital	Community		
		Public	NGO		Public	NGO	
**Total New Admissions**	**524**	**302**	**86**	**554**	**121**	**21**	**1608**
Not eligible	N/A	79	9	N/A	15	0	103
^a^Happy to be contacted: No	N/A	183	33	N/A	35	0	251
Happy to be contacted: Yes	N/A	40	44	N/A	71	21	176
First Conversations: No	414	13	22	296	27	0	772
First Conversations: Yes	110	27	22	258	44	21	482
ACD Completed	4 (3.6%)	11 (40.7%)	15 (68.1%)	3 (1.2%)	36 (81.8%)	16 (76.1%)	85 (17.6%)
		26 (53%)			52 (80%)		
Number of conversations (mean)	11 (2.75)	47 (4.27)	66 (4.4)	12 (4)	94 (2.6)	52 (3.3)	282 (3.3)
Duration (minutes)	690	2965	3885	490	9917	4829	22,776
Mean (minutes)	62.72	63.09	58.86	40.83	105.5	92.86	80.77
Conversation cards with ACD completion	4	11	15	3	36	16	85
Conversation cards without ACD completion	1	4	4	5	14	7	35

^a^Potential participants at community sites were initially contacted via phone to engage if they were happy to be contacted by an ACP RN

### Reasons for lack of engagement on ACP with patients in hospital and community settings

The details of reasons provided by those who met the inclusion criteria but who declined to have the first conversation with ACP RNs are provided in Table 5. Total number of reasons exceeds the total number of patients, given that the ACP RNs tried to engage with patients on more than one occasion, for example, when the patient was away for a scan or a dialysis. In hospital settings in both LHDs, almost 60% (*n* = 898) of patients were not engaged in the first conversation because patients were too unwell, not interested, asleep, away at scan or dialysis, experienced cognitive decline or acute episode of mental health issue, had hearing impairment, felt too much going on emotionally and physically, needed time to think about, and will do it at home. In both LHDs, the most common reason for decline of NACP service in community setting was that patients (26%, *n* = 67 in LHD1 Public; 64.3%, *n* = 27 in LHD1 NGO; 49%, *n* = 29 in LHD2 Public) were not interested.

**Table 5:** The reasons for not being able to engage patients in ACP in hospital and community settings

Reasons	**Hospital settings**			
	LHD1 (*n* = 414/524)		LHD2 (*n* = 296/554)	Total (710/1078)
1. SDM know my wishes; No SDM in hospital	23 (3.2%)		56 (6.9%)	79 (5.2%)
2. Patient: were too unwell, not interested in, asleep, away at scan or dialysis, experienced cognitive decline or acute episode of mental health issue, had hearing impairment, felt too much going on emotionally and physically, need time to think about, and will do it at home	416 (59.5%)		482 (59.3%)	**898 (59.4%)**
3. Visited by Doctor	9 (1.3%)		34 (4.2%)	43 (2.8%)
4. Visited by Nurse	11 (1.6%)		30 (0.4%)	41 (2.7%)
5. Visited by Allied Health Professional	18 (2.6%)		20 (0.3%)	38 (2.5%)
6. Visited by family and friends	5 (0.7%)		39 (4.8%)	44 (2.9%)
7. Discharged or transferred or deceased	**159 (22.7%)**		41 (5%)	200 (13.2%)
8. Ran out of time	34 (4.9%)		2 (0.2%)	36 (2.4%)
9. Already have ACD (done with solicitor, not in hospital medical record)	15 (2.1%)		21 (2.6%)	36 (2.4%)
10. Not-For-Resuscitation order in place	9 (1.3%)		88 (0.9%)	97 (6.4%)
Total	699 (100%)		813 (100%)	1512 (100%)
Reasons	**Community Settings**			
	LHD1		LHD2^a^	Total
	Public (*n* = 257)	NGO (n = 42)	Public (*n* = 50)	349
1. Not eligible (No chronic condition, No capacity)	79 (31%)	9 (21.4%)	15 (25.4%)	103 (28.8%)
2. Not interested in	**67 (26%)**	**27 (64.3%)**	**29 (49%)**	**123 (34.4%)**
3. Feels too young	3 (1.2%)	0	0	3 (0.8%)
4. Transferred to or in hospital	5 (1.9%)	0	0	5 (1.4%)
5. Too busy	1 (0.4%)	0	0	1 (0.3%)
6. Home Care Service closed	95 (37%)	0	0	95 (26.5%)
7. Too unwell or passed away	5 (1.9%)	3 (7.1%)	0	8 (2.2%)
8. Have all sorted (not sighted)	2 (%)	3 (7.1%)	13 (22%)	18 (5.0%)
9. Palliative care	0	0	2 (3.4%)	2 (0.6%)
Total	257 (100%)	42 (100%)	59 (100%)	358 (100%)

LHD2^a^: All clients (*n* = 21) in NGO community setting in LHD2 have accepted ACP Service (See Table 4). *SDM* Substitute Decision Maker, *ACD* Advance Care Directive

## Discussion

ACP conversations were normalised by offering these to all new admissions. A total of 85 ACDs completed in this study were legally binding and the location of those ACDs were known to patients’ SDMs and General Practitioners (GPs) who will act on their behalf when the time comes. This study adds new evidence on how long it took to completion of ACD by an individual (average 80.77 min per conversation, 3.3 conversations with the ACP RNs, and 4.44 h of the ACP RNs time spaced around 4 to 6 weeks apart). This is similar to the findings in another study [32] which reported that the inpatients (*n* = 63) in Melbourne, Australia had a median of two conversations with a median duration of discussion just over 1 h. Older patients, and patients with CAD, Cancer, and Frailty were more likely to have completed ACD, compared with patients without these conditions. Patients with Dementia, CKD and CHF were less likely to have completed ACDs compared to those without these conditions. The control sites that did not have an ACP RN kept their consistently low number of ACD completion.

Earlier studies emphasised the importance of appointing a substitute decision maker especially for older people at the end of life, and indicated that one may be reluctant to document ACDs but be likely to have someone in mind who they would like to make decisions for them [33–35]. However, various terms appear with variations in jurisdictional legislation, for example, an EG in Australia and ‘Lasting Power of Attorney for Health and Welfare’ in the UK [36], and ‘a legal proxy’ in German [37], even with a consensus that ACP includes ACDs and the appointment of a proxy decision maker [38]. The challenges with the lack of uniform term across the world include misunderstanding of the responsibility and legality, and underreporting or misreporting of the prevalence of EG [35, 39]. This study specified the definition of an EG in Australian context (See Supplementary file 2) to increase clarity and accuracy of the results. In contrast with the previous predictions [33–35], the number of people who appointed an EG intervention sites made only a small increase from 33 (5.62%) to 37 (7.68%) whilst the number of people who documented ACDs increased from five to 85. A low uptake of appointment of an EG is consistent with previous studies which reported 6% of 115 people from Culturally and Linguistically Diverse Background (CALD) [35]. A higher uptake of ACDs may indicate that when people were given an opportunity and facilitated by ACP RNs, they would rather documenting ACDs than appointing an EG which would require a legal process. Given the variations in the definition of EG and the fact that people practise autonomy within their social and cultural context [40], further investigation is warranted.

### The future of ACP promotion and roles for health professionals in hospital settings

The ACP RNs in the hospital sites (*n* = 1078) had access to many more eligible patients than the community ACP RNs (*n* = 530). However, there was very little difference in the number of ACDs completed at hospital sites between the pre and post which was four to seven in intervention and four to one in control groups respectively. This finding is contrary to another study [31] that reported a 54% (*n* = 63) completion rate of ACDs in a hospital setting. In this study, most patients (60%) in hospital sites were unable to be engaged in the first conversation for reasons identified above, despite the presence of the ACP RNs on the ward. It was unrealistic to expect an acutely unwell patient in a hospital ward to have the energy and capacity to discuss very sensitive, important and in-depth topics for such a long time period without disruption. This finding clearly indicates hospital setting may not be an ideal site for ACP discussion and ACD completion. This finding also raises the questions, if and to what extent should ACP be promoted in hospital setting? What will be the roles for healthcare professionals in hospital settings? The findings from this study suggests that healthcare professionals in hospital setting can distribute the brochure about ACP and make a referral of those who are interested to ACP RNs in community to follow up. This finding also has an implication for policy makers and healthcare professionals in the provision of ACP service planning and delivery how to promote ACP in an integrated way between hospital and community settings.

### Why community?

While Detering et al. [41] questioned implementation of ACP into community due to low numbers and quality of ACDs in their study, the ACP RNs in the community sites in this study were much more successful at promoting ACP, leading to a significant increase (from 0 to 26 (2600% increase) in LHD1 and from one to 52 (5100% increase) in LHD2 of the number of ACDs completed. Out of 85 ACDs completed 78 (92%) ACDs were completed by people in the community sites. This study has established new evidence that the ACP service delivered by specially trained ACP RNs was more feasible and effective in uptake of ACP and completion of ACDs, and was more effective in community-based health service, including both public and NGO sector, compared to hospital settings.

There are a few likely factors that lead to this significant increase in community sites compared to the hospital wards. First, patients visited by community nurses were less likely to have an acute illness that would prevent them from wanting to talk with the ACP RNs. Second, the other community nurses at each site (nurses other than the ACP RN) who introduced NACP service were able to act as a secondary screening/promotion tool. Third, being able to engage the patients in discussion about ACP in their own home led to much more productive conversations. These sessions were also more easily attended by the patient’s family. Taken together, the study findings indicate that a nurse specially trained to promote ACP through community health care services can increase the number of people engaging in ACP and completing ACDs. This is consistent with the emerging literature [19, 27, 42], however, further research is warranted.

### How do we ensure concordance of care?

Those 85 participants who completed ACDs documented their values in life, goals for health care, and future health care preferences including the preferred place for death. In fact, during the intervention period, two patients in LHD1 who previously made frequent hospitalisations completed their ACDs and peacefully passed away with their loved ones around at home as they wished with the support of their GPs and palliative care service team. This evidence indicates what it takes to execute the documented wishes in ACDs. Detering et al. [32]. emphasised that the documented wishes should be shared and managed by multidisciplinary health professionals. This study elaborates on the need for and extends involvement of stakeholders of ACP which warrants inclusion of GPs, Palliative Care service team, organ donation coordinators, Ambulance Officers, and Medical Officers and RNs in hospital who then must respect the documented wishes in ACD and should integrate and coordinate appropriate services to ensure concordance of care.

### Promotion of ACP to those (25%) who are not interested

Despite policies, legislation (not all but in some jurisdictions in Australia), numerous resources and individualised conversations facilitated by specially trained RNs, this study established new evidence that in average 46% of people (26% in LHD1 public sector, 64.3% in LHD1 NGO sector, and 49% in LHD2 Public sector) with chronic conditions in community were not interested in ACP. This finding raises important questions on expectations about the achievement of the maximum level of uptake that people will engage in ACP and to what extent ACP should be promoted to those who show no interest on the process. Healthcare professionals are challenged with the dilemma of if and how we should reach out to people to help them see any personal benefit of engaging with ACP. ‘Simply not interested in’ might have been the natural response from people in the community to those with another ‘sales pitch’. This warrants further investigation.

### Limitations

The intervention was implemented in hospital and community settings in two metropolitan LHDs, which means the feasibility and efficacy of the NACP service by RN ACP facilitators will not be generalisable to residential aged care settings, rural and remote settings, and other LHDs. Given the six-month (including 2 weeks of training) intervention period, it was not possible to determine the concordance of care between care preferences in ACDs and care that were executed, other than two participants. A longer period of intervention and data collection is recommended to determine more definite outcomes along the process of ACP. Given the established feasibility and efficacy of NACP service, it can be translated and scaled to other community sites.

## Conclusion

ACP has been the quest of dedicated and committed healthcare professionals worldwide who encounter heartbreaking and undignified deaths in their daily practice. The challenges with ACP lie in each step of ACP processes from initiation, discussion, documentation, and storage to execution. The intervention proposed in this study addressed those challenges in each step. This study provides evidence of the feasibility and efficacy of NACP service for people with chronic conditions and adds new evidence that suggests NACP facilitated by RNs was more effective in the community than the hospital setting. ACP, if done properly, takes time and appropriate training, but helps people die in a way that is consistent with their wishes. Further investigations are warranted to answer the questions posed above.

## Supplementary Information

**Additional file 1.**

**Additional file 2.** The definitions of ACD and EG.

## Data Availability

All data relevant to the primary outcomes are included in this published article.

## Abbreviations

ACD: Advance Care Directives
ACP: Advance Care Planning
CAD: Coronary Artery Disease
CKD: Chronic Kidney Disease
CHF: Congestive Heart Failure
COPD: Chronic Obstructive Pulmonary Disease
EG: Enduring Guardian
FR: For-Resuscitation
GP: General Practitioner
LHD: Local Health District
NFR: Not-For-Resuscitation
RN: Registered Nurse
SDM: Substitute Decision-Maker
