# Supplementary figures and images for: Efficacy of Normalisation of Advance Care Planning (NACP) for people with chronic diseases in hospital and community settings: a quasi-experimental study
Source: BMC Health Serv Res. 2021 Sep 1;21:901. doi: 10.1186/s12913-021-06928-w (PMC8408987; doi:10.1186/s12913-021-06928-w)

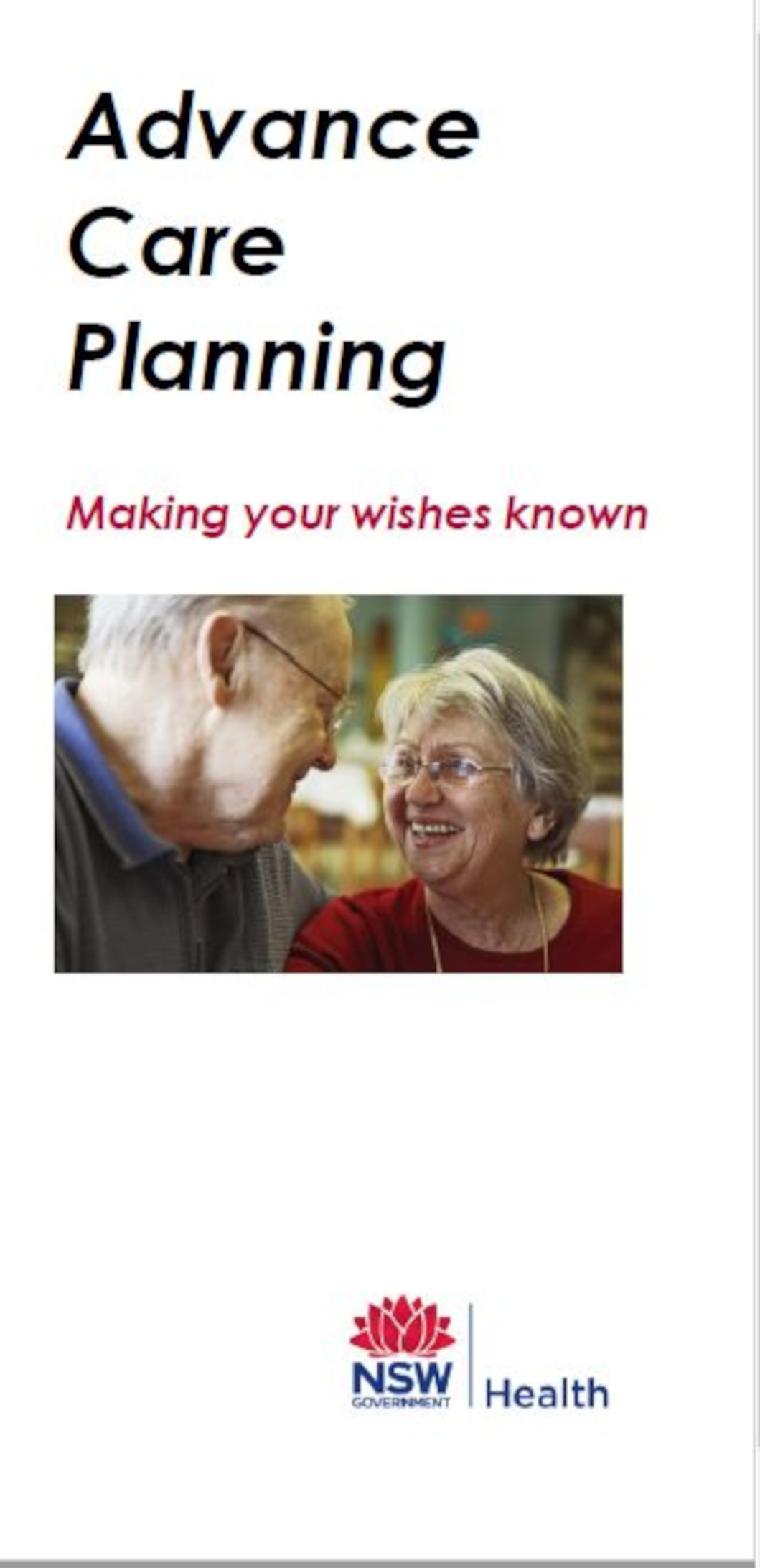

Supplement: Supplementary file 1 — Additional file 1. [file 12913_2021_6928_MOESM1_ESM.zip › 20210715 Supp file 1a. ACP Making your wishes known_300dpi.JPG]

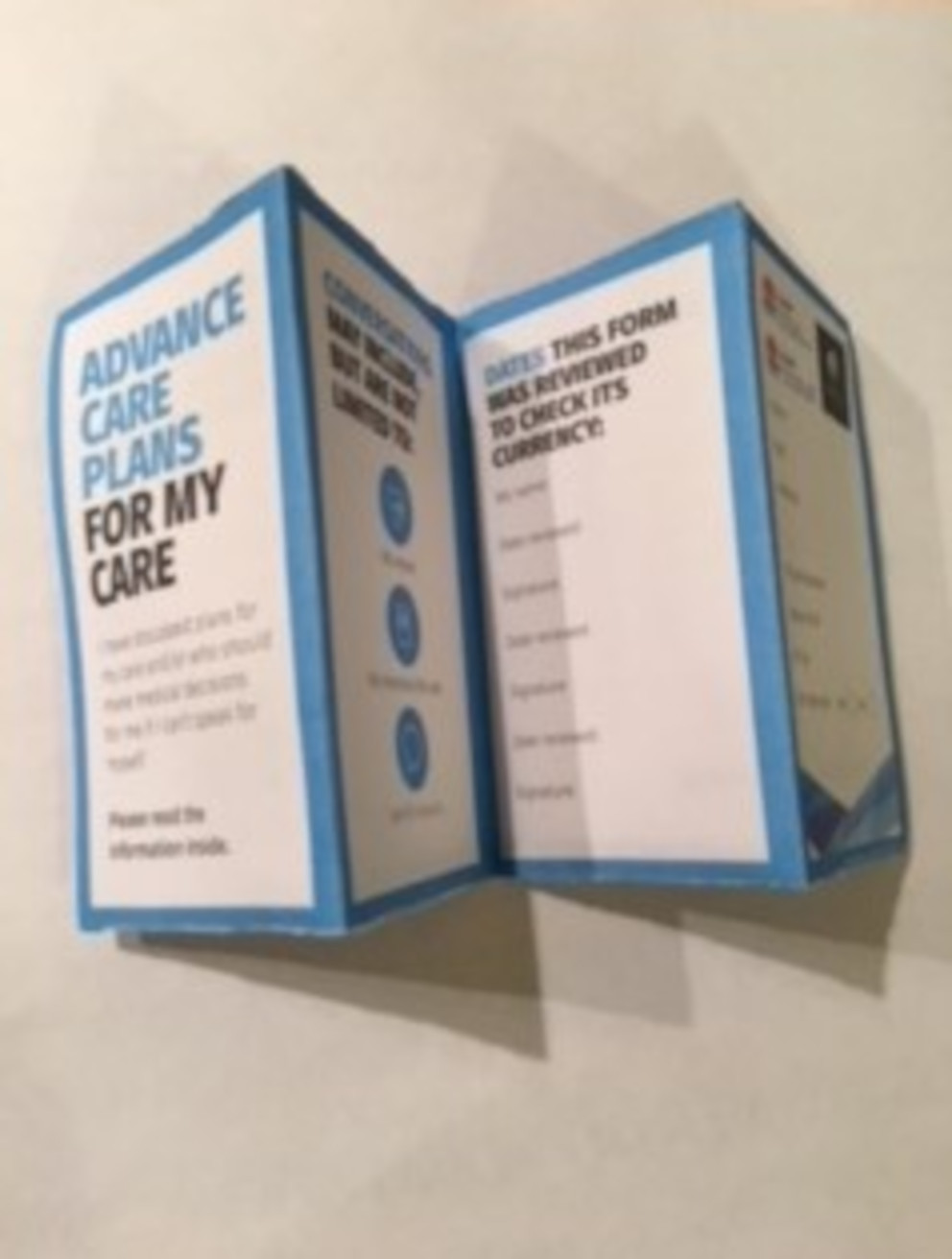

Supplement: Supplementary file 1 — Additional file 1. [file 12913_2021_6928_MOESM1_ESM.zip › 20210715 Supp file 1c. Conversation card folded_300dpi.JPG]

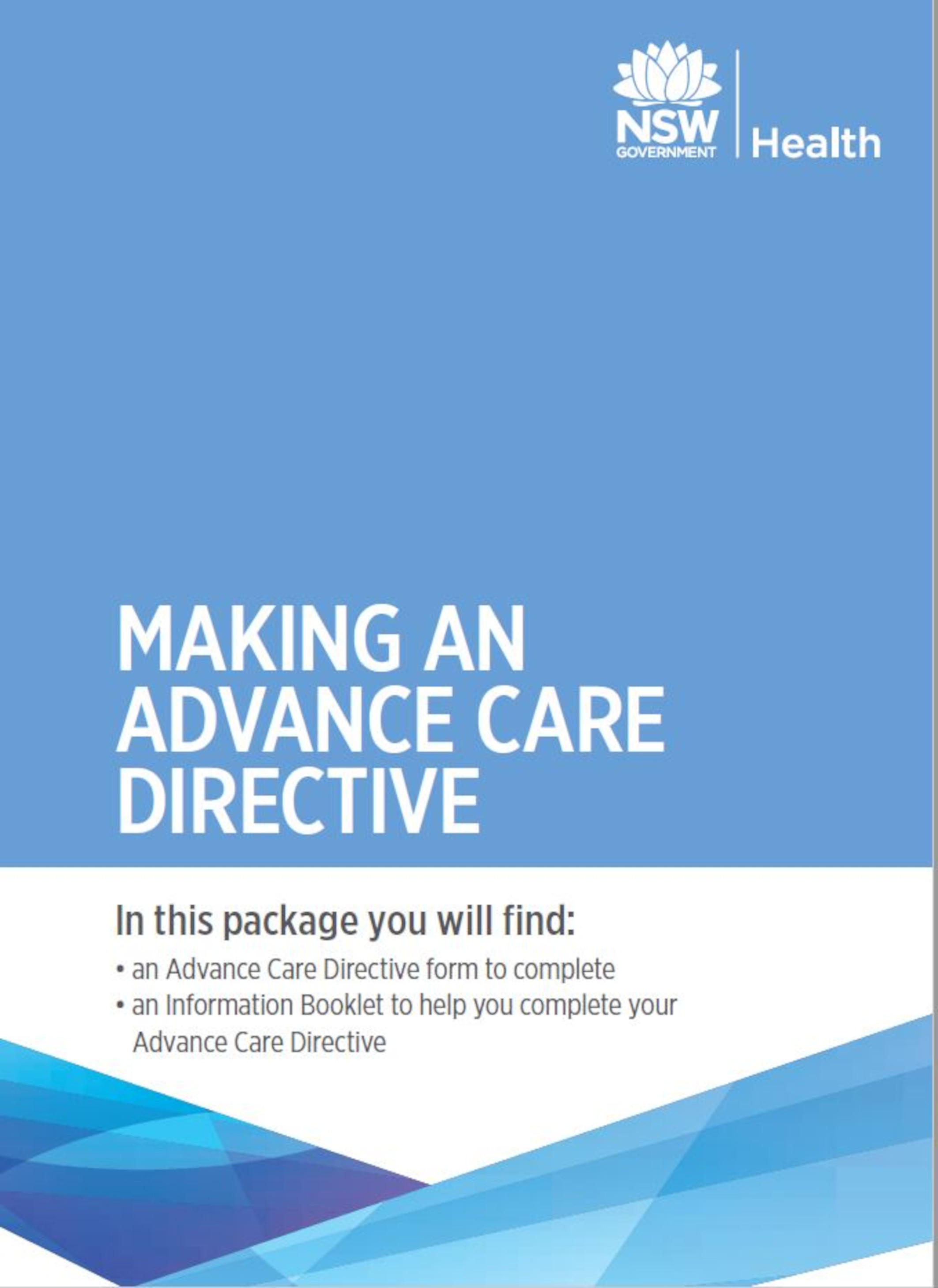

Supplement: Supplementary file 1 — Additional file 1. [file 12913_2021_6928_MOESM1_ESM.zip › 20210715 Supp file 1d. NSW Making an ACD_300dpi.JPG]
