# Supplementary material for: Efficacy of Normalisation of Advance Care Planning (NACP) for people with chronic diseases in hospital and community settings: a quasi-experimental study
Source: BMC Health Serv Res. 2021 Sep 1;21:901. doi: 10.1186/s12913-021-06928-w (PMC8408987; doi:10.1186/s12913-021-06928-w)
Supplement: Supplementary file 2 — Additional file 2. The definitions of ACD and EG. [file 12913_2021_6928_MOESM2_ESM.docx]

**Title: Efficacy of Normalisation of Advance Care Planning (NACP) for people with chronic diseases in hospital and community settings: a quasi-experimental study**

**Authors**

Sarah Jeong^1^*, Peter Cleasby^2^, Se Ok Ohr^3^, Tomiko Barrett^4^, Ryan Davey^5^ and Christopher Oldmeadow^6^

^1^*School of Nursing and Midwifery, University of Newcastle, 10 Chittaway Road. Ourimbah. NSW, 2258, Australia. [Sarah.Jeong@newcastle.edu.au](mailto:Sarah.Jeong@newcastle.edu.au)

^2^Division of Aged, Subacute and Complex Care, PO Box 6088 Long Jetty, Central Coast Local Health District. NSW 2261, Australia. [Peter.Cleasby@health.nsw.gov.au](mailto:Peter.Cleasby@health.nsw.gov.au)

^3^Hunter New England Nursing and Midwifery Research Centre, James Fletcher Campus, Gate Cottage, 72 Watt St, Newcastle, Hunter New England Local Health District. NSW 2300, Australia. [Seok.Ohr@hnehealth.nsw.gov.au](mailto:Seok.Ohr@hnehealth.nsw.gov.au)

^4^ Dept of Aged Care Services, Wyong Hospital PO Box 4200 Lakehaven, Central Coast Local Health District. NSW 2263, Australia [Tomiko.Barrett@health.nsw.gov.au](mailto:Tomiko.Barrett@health.nsw.gov.au)

^5^School of Nursing and Midwifery, University of Newcastle, 10 Chittaway Road. Ourimbah. NSW, 2258, Australia. [r.davey@uon.edu.au](mailto:r.davey@uon.edu.au)

^6^Hunter Medical Research Institute, Lot 1, Kookaburra Circuit, New Lambton Heights, Newcastle 2305, NSW, Australia. [Christopher.Oldmeadow@hmri.org.au](mailto:Christopher.Oldmeadow@hmri.org.au)

***Correspondence: Associate Professor Sarah Jeong**

**Additional file 2. The definitions of ACD and EG**

- ACD: a legally binding document made by a legally capable person about the person’s specific wishes and preferences for future care. This includes treatments they would accept or refuse if they had a life-threatening illness or injury, their values in life and goals of care [30]. For an ACD to have sufficient authority to act on, in other words, to be legally binding, four conditions of relevance, specificity, competence and currency should be satisfied [5, 8].
- An EG: an individual(s) who is legally appointed by the person and who can legally make decisions on behalf of the person about the person’s medical and dental care, if the person loses capacity to make decision [30].
